# Chemical Composition, Toxicity and Vasodilatation Effect of the Flowers Extract of *Jasminum sambac* (L.) Ait. “G. Duke of Tuscany”

**DOI:** 10.1155/2012/471312

**Published:** 2012-03-26

**Authors:** Phanukit Kunhachan, Chuleratana Banchonglikitkul, Tanwarat Kajsongkram, Amonrat Khayungarnnawee, Wichet Leelamanit

**Affiliations:** ^1^Department of Biochemistry, Faculty of Pharmacy, Mahidol University, 447 Sri Ayudthya, Rajathevi, Bangkok 10400, Thailand; ^2^Pharmaceutical and Natural Products Department, Thailand Institute of Scientific and Technological Research (TISTR), Klong 5, Klong Luang, Pathum Thani 12120, Thailand

## Abstract

Phytochemical analysis of the ethanolic Jasmine flower extract of *Jasminum sambac* (L.) Ait. “G. Duke of Tuscany” revealed the mixtures of coumarins, cardiac glycosides, essential oils, flavonoids, phenolics, saponins, and steroids. However, alkaloids, anthraquinones, and tannins were not detected. By intravenous injection at a single dose of 0.5 mL/mouse (15 mg) of the flower extract, no systemic biological toxicity demonstrated in ICR mice was observed. In Wistar rats, the LD_50_ of the extract was higher than 5,000 mg/kg BW by oral administration. Vasodilatation effect of the 95% ethanolic extract on isolated aortic rats was also investigated. Compared with the control group, the Jasmine flowers extract in 0.05% DMSO clearly reduced tonus of isolated endothelium thoracic aortic rings preconstricted with phenylephrine (10^−6^ M), as a dose-dependent manner. Nevertheless, this pharmacological effect disappeared after the preincubation of the rings with atropine (10^−6^ M) or with N^*ω*^-nitro-L-arginine (10^−4^ M). These are possibly due to the actions of the active components on the vessel muscarinic receptors or by causing the release of nitric oxide.

## 1. Introduction

Jasmine (*Jasminum*) is a genus containing approximately 600 species of small trees and vines in the Family Oleaceae. These glabrous twining shrubs are widely cultivated in gardens and easily found in forests throughout tropical Asia and warm temperate regions in Europe and Africa. Their flowers and leaves have been well recognized for multipurpose uses. For instance, the flowers have been utilized as traditional medicines in Asia to treat many diseases including diarrhea, fever, conjunctivitis, abdominal pain, dermatitis, asthma, abscess, breast cancer, uterine bleeding, and toothache. In China, the leaf parts are used for the treatment of quadriplegia gall, dysentery, and bellyache. According to its high medicinal value, *Jasminum sambac* is one of the most cultivated species in many countries in Asia including Thailand. Its phytoconstituents contain iridoidal glycosides [1], linalyl 6-*O*-malonyl-**β**-D-glucopyranoside, benzyl 6-*O-*β**-D-xylopyranosyl-**β**-D-glucopyranoside (**β**-primeveroside), 2-phenylethyl **β**-primeveroside, 2-phenylethyl 6-*O-*α**-L-rhamnopyranosyl-**β**-D-glucopyranoside (**β*-*rutinoside) [2], dotriacontanoic acid, dotriacontanol, oleanolic acid, daucosterol, and hesperidin [3]. The volatile constituents consist of benzyl acetate, indole, E-E-**α**-farnesene, Z-3-hexenyl benzoate, benzyl alcohol, linalool, and methyl anthranilate [4]. Although the whole parts of the plant are employed and prescribed in folk medicines, only two pharmacological studies of *J. sambac* have been reported. The flower displayed the efficacy to suppress puerperal lactation [5] and the essential oil was determined to possess antibacterial activity [6]. The objective of the present study was to examine the toxicity and vasodilatation activities of *J. sambac* (L.) Ait. “G. Duke of Tuscany” (locally called Ma-li-son), a local variety commonly found in Thailand. The vasodilation effect of the ethanolic extract was first reported using isolated thoracic aortic rings. The phytochemical composition and the toxicity were also assessed.

## 2. Materials and Methods

### 2.1. Chemicals

Phenylephrine chloride (PE), N^*ω*^-nitro-L-arginine (L-NA), atropine sulfate, acetylcholine chloride (Ach), rutin hydrate, oleuropein, kaempferol disaccharides, and quercetin were purchased from Sigma-Aldrich Chemical, USA. All other chemicals used were of analytical grade.

### 2.2. Animals

Wistar rats (*Rattus norvegicus*) and ICR mice (*Mus musculus*) used were obtained from the National Laboratory Animal Center, Salaya, Mahidol University, Nakhon Pathom, Thailand. All animals were acclimatized for 1 week before starting of the experiments in controlled environmental conditions (25 ± 1°C) with a 12 h light/dark cycle and allowed access to standard food and tap water *ad libitum*. Animal welfare was under control by IACUC of the Thailand Institute of Scientific and Technological Research (TISTR).

### 2.3. Plant Material and Extraction

The flowers of *J. sambac* (L.) Ait. “G. Duke of Tuscany” were collected from Nakhon Pathom province, Thailand, in March 2009. The specimen voucher was TISTR no. 160309, and the samples were deposited at TISTR. Approximately 3.1 grams of the flowers were dried at 50°C for 42 h, powdered, and macerated in 18 mL of 95% ethanol at room temperature overnight. After elution, the flower residues were repeatedly macerated with equal volume of ethanol overnight and eluted again. The ethanol elutes were combined, filtered through Whatmann filter paper no. 42, and evaporated under reduced pressure at 50°C. The semisolid light yellow materials were stored in desiccators until used. Percentage yield of the extract was 17.68% yield (w/w). The extract was dissolved in 0.05% dimethxyl sulfoxide (DMSO) for* in vitro* experiments and in 1% Tween or 1% gum tragacanth for* in vivo *studies. 

### 2.4. Thin Layer Chromatography (TLC) Analysis

The ethanolic flower extract was screened for the different classes of compounds by thin layer chromatography using silica gel 60 F254 (Merck, Darmstadt, Germany) plates of 0.25 mm thickness. Briefly, the extract was dissolved in methanol while the development of plates was carried out with different mobile phase systems using appropriate reagents as standard makers (Table 1). After development, the plates were sprayed with the following solvents and reagents for detection of the respective classes of compounds: 0.5% anisaldehyde in sulfuric acid, glacial acetic acid, and methanol 5 : 10 : 85 v/v and heating at 105°C for 5–10 min; 10% solution of antimony trichloride in chloroform and heating at 105°C for 5-6 min (for phenolics/tannins); 1% methanolic diphenylboryloxyethylamine, followed by 5% ethanolic polyethylene glycol 4000 (PEG) (for flavonoids), 5% ethanolic potassium hydroxide (for anthraquinones/coumarins), Dragendorff's reagent (for alkaloids), DPPH reagent (for antioxidants), the Liebermann-Burchard reagent (for steroids), and the Kedde reagent (for cardiac glycosides). Reagents were prepared according to Wagner and Bladt, 1996 [7]. Saponins were detected by observing froth formation by the extract in a test tube after regular shaking. The colored, double-bonded, and fluorescent compounds were detected on the silica gel plates under day light, UV light 254 nm, and UV light 365 nm, respectively.

### 2.5. HPLC Analysis

For HPLC analysis, the flower extract (1.0 g) was dissolved in 100 mL of water in an ultrasonic bath for 15 min. The solution was partitioned three times with 20 mL of hexane. The aqueous layer was also partitioned three times with 20 mL of ethyl acetate and then three times with 20 mL of butanol. After the partitioned samples were dried in an evaporator, the obtained solid samples were dissolved in 10 mL of 60% methanol and were used for HPLC analysis. HPLC analysis was performed on a Waters apparatus (Waters 2695 separations Module, Alliance and Waters 2996 Photodiode Array Detector) using a Phenomenex Luna C18, 5 *μ*m, 250 × 4.60 mm column (Waters). The mobile phase was 0.05% phosphoric acid (pH 2.3) and methanol. The flow rate was 0.5 mL/min, the injection volume was 20 *μ*L, and the run times was 45 min. UV-visible spectra were recorded in the range of 190–450 nm, and chromatograms were acquired at 280 and 350 nm. Four standard flavonoids (rutin, kaempferol disaccharide, quercetin, kaempferol) and one cardiac glycoside (oleuropein) were dissolved in methanol [8].

### 2.6. Systemic Biological Reactivity Test

Ten male ICR mice weighing between 22–25 g were divided into two groups. Group I received no treatment. Group II mice individually received 0.5 mL (15 mg) of the extract via intravenous injection using a 26-gauge needle. The treated animals were investigated immediately after injection and at 4, 24, 48, 72 h after injection. The body weights of all animals were measured at Days 8 and 15. At Day 15, the mice were killed by cervical dislocation, and internal organs were weighed and collected for pathological examination. 

### 2.7. Acute Oral Toxicity Test

Twenty Wistar rats (10 animals per sex) weighing 200–250 g were divided into two groups, and both groups were fed under standard laboratory conditions. All rats were kept overnight fasting prior to Jasmine extract administration. One group as control received 1% gum tragacanth, while the treated group received the flower extract orally at a single dose of 5,000 mg/kg. After 3 h of the administration, food was allowed. The rats were observed individually at least once within the first 24 h (with special attention to the first 4 h) and daily thereafter for a period of 15 days. The body weights of the rats were measured at Days 8 and 15. At Day 15, the rats were terminated and the weights of the internal organs were determined.

### 2.8. Vasodilatation Effect Test

Each rat was killed by cervical dislocation. Its thorax was opened, and the aortic vessel was removed from fat and connective tissues and kept in a Petri dish containing Krebs' solution. Two adjacent aortic rings of 3-4 mm in length were cut. In only one ring, endothelium was removed mechanically by gently rubbing the intimal surface of the vessel using the method as previously described [9]. The organ bath contained the Krebs-Henseleit solution (NaCl 119, KCl 4.7, CaCl_2_ 2.5, MgSO_4_·7H_2_O 1.0, KH_2_PO_4_ 1.2, NaHCO_3_ 25.0, and glucose 11.1 mM). This solution was maintained at 37°C and continuously bubbled with 95% O_2_ and 5% CO_2_. The two rings were individually suspended horizontally between two stainless steel hooks in a 20 mL organ bath containing Kreb's solution. One of the hooks was fixed to the bottom, and the other was connected to a force displacement transducer that connected to a MP100 (BIOPAC System, Inc., Model MP100 WSW) for the isometric tension record. The stabilization period was of 45 min under a resting tension of 1.0 g and the solution was changed every 15 min to prevent the accumulation of metabolites. After equilibration, the rings were preconstricted with 1 × 10^−6^ M phenylephrine (PE) until the responses curve reached plateaus (5–8 min), and dilator responses to 1 × 10^−5^ M acetylcholine (Ach) were detected. The absence of the relaxation to acetylcholine was taken as evidence that the vessel segment was functionally denuded of endothelium, and at least 70% vasodilatation to acetylcholine for the endothelium-intact thoracic aorta rings was observed. 

#### 2.8.1. Effect of the Jasmine Flower Extract on PE-Induced Tonus in the Endothelium-Intact or Endothelium-Denuded Thoracic Aorta Rings [10]

After 45 minutes re-equilibration, the rings with and without endothelium intact were pre-contracted with 1 × 10^−6^ M PE for 20 minutes. After several washings and re-equilibration for 30 minute, five different concentrations of the flower extracts (50, 100, 200, 300, and 400 *μ*g/mL) were added 5 min and the rings were incubated with PE for another 20 min. The contractions were measured by comparing the developed tension before and after the addition of the extract and expressed as percentage of contraction from induced tonus.

#### 2.8.2. Effect of the Extract on PE-Induced Tonus in the Endothelium-Intact Thoracic Aorta Rings with Atropine and N^*ω*^-Nitro-L-arginine [10]

After 45 min re-equilibration, the endothelium-intact aortic rings were pre-constricted with 1 × 10^−6^ M PE for 20 min. After several washings and re-equilibration for 30 minutes, the rings were exposed to atropine (1 × 10^−6^ M) or N^*ω*^-nitro-L-arginine (L-NA), a nitric oxide synthase inhibitor (1 × 10^−4^ M), for 5 minutes. Then, the same serial concentrations of the extracts were added, and the percentage of the contractions before and after the addition of the extracts was determined as described above.

### 2.9. Statistical Analysis

The results are expressed as means ± standard error means (SEMs). Student's *t*-tests or one-way ANOVA were used for statistical analysis. In all cases, *P* values < 0.05 were considered statistically significant.

## 3. Results

### 3.1. Phytochemical Evaluation

Phytochemical analysis of the ethanolic extract displayed that antioxidants, coumarins, cardiac glycosides, essential oils, flavonoids, phenolics, saponins, and steroids were investigated. Other compounds such as alkaloids, anthraquinones, and tannins were not found (Table 2).

### 3.2. Systemic Biological Reactivity Test

At a dose of 15 mg (0.5 mL/mouse) of the extract by tail vein injection, no toxic signs or mortality was observed. Compared to the control group, there was no statically significant difference (*P* < 0.05) in the body and organ weights of the mice between the two groups (Table 3). 

### 3.3. Acute Oral Toxicity Test

The extract at a single dose of 5,000 mg/kg BW did not cause any sign of toxicity in rats. For both sexes, there was no statically significant difference (*P* < 0.05) in the body and organ weights of rats between the control and treated groups (Tables 4 and 5). In addition, the rats of both groups displayed normal behaviors.

### 3.4. Vasodilatation Effect Test

**Figure 1:**
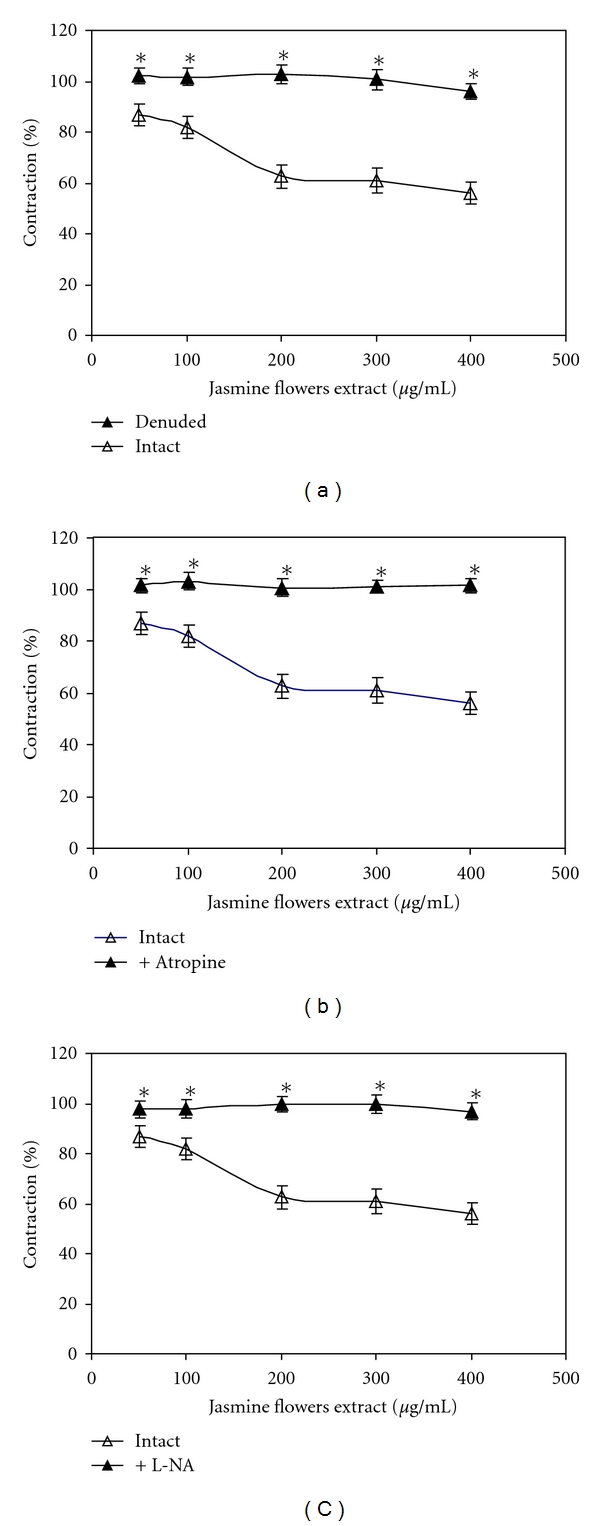
Dose-response curves for the vasodilation effects of the ethanolic Jasmine flower extract on the preconstricted thoracic aorta rings. Results are presented as means ± SEM (*n* = 6), (Student's *t*-test, *P < 0.05). (a) Endothelium intact (*▵*), endothelium denude (▲). (b) Endothelium intact (*▵*), with atropine (▲). (c) Endothelium intact (*▵*), with L-NA (▲).

PE at 1 *μ*M produced a steady-state contraction in the aortic rings with or without endothelium. As shown in Figure 1, the extract (50–400 *μ*g/mL) caused vasodilation of the endothelium-intact thoracic aorta ring pre-constricted with phenylephrine in a dose-response manner. However, this effect disappeared after preincubation of the aortic rings with atropine (1 × 10^−6^ M), L-NA (1 × 10^−4^ M) or by removal of the vascular endothelium. 

## 4. Discussion

*Jasminum sambac* is one of the most well-famous fragrant plants worldwide and has been prescribed in folk medicines in many countries according to its multipurpose actions. In addition, Jasmine tea is the most famous scented tea in many countries including China, Japan, and Thailand. Nevertheless, the chemical constituents and pharmacological activities of *J. sambac* have been rarely reported. 

To date, the flower of *J. sambac* was reported to contain the mixtures of dimeric and trimeric iridodial glycosides (molihuasides A–E) and glycosidic aroma precursors [1, 2]. Recently, Edris et al. reported that the main volatile constituents from the flower extract were benzyl acetate, indole, E-E-**α**-farnesene, Z-3-hexenyl benzoate, benzyl alcohol, linalool, and methyl anthranilate [4]. Our TLC analysis indicated that the ethanolic flower extract contained the mixtures of coumarins, cardiac glycosides, essential oils, flavonoids, phenolics, saponins, and steroids. However, alkaloids, anthraquinones, and tannins were not detected. Data from HPLC analysis also revealed high content of flavonoid mixtures (data not shown). Therefore, the various therapeutic actions used in different traditional medicines are definitely attributed to the mixtures of active ingredients in the Jasmine flower. 

Although this Jasmine species is used as a principal ingredient in many traditional medicines or in tea industries, its toxicity has never been documented elsewhere. At a high dose of the flower extract via intravenous injection (15 mg/mouse), no biological reactivity in male ICR mice was observed. For acute toxicity, the LD_50_ was higher than 5,000 mg/kg in both sexes of Wistar rats. In both toxicity assays, there was no statically significant difference (*P* < 0.05) in the body and organ weights between the control and treated groups. In gross examinations, the individual internal organs of the treated and the control groups displayed no significant difference. Additionally, the rats of both groups had normal behavior. For the liver function test, the activities of aspartate aminotransferase (AST) and alanine aminotransferase (ALT) were determined and there were no enzymatic difference between the control and treated groups (data not shown). As confirmed by traditional use for thousand years in many countries, the flower of *J. sambac* is therefore safe for general utilization in medicines or food industry. 

The flower extracts of *J. sambac* displayed suppression of puerperal lactation and antibacterial activity. Here, we first described that the flower extract of *J. sambac* possessed vasodilation activity. As shown in Figure 1(a), the vasodilation effect of the Jasmine flower extract was mediated by endothelial cells in the aortic vessel. The flower extract at a dose of 400 *μ*g/mL reduced the contraction to lower than 43% of the maximal contraction. This result suggests that the vasorelaxation effect of the ethanolic* J. sambac* flowers extract is endothelium dependent. It has been reported that the vasorelaxant property of most plant extracts was from flavonoids [11]. Thus, the vasodilation activity should be attributed to the high content of flavonoid mixtures found in the Jasmine flower extract. 

Preincubating the thoracic aortic rings with atropine (10^−6 ^M), a muscarinic receptor antagonist completely blocked the relaxant activity of the extract (Figure 1(b)). The pharmacologically relaxant effect of the flower extract was further determined. Pretreatment of endothelium-intact aorta with L-NA (1 × 10^−4^ M) also reduced the vasodilation effect of the extract to an extent equivalent to the effect in endothelium-denuded aorta (Figure 1(c)). Nowadays, the mechanism of nitric oxide (NO) and the function of endothelial cells in the relaxation of arteries are well described. NO is a potent vasodilator synthesized in the endothelium by NO synthase and causes vascular relaxation [12–14]. The result from the present study suggests that the Jasmine flower extract may exert its endothelium-dependent relaxation activity by stimulating the nitric oxide release from the vascular endothelium via muscarinic receptors.

## 5. Conclusion

The Jasmine flowers extract at a dose of 0.5 mL/mouse (15 mg) by intravenous injection in male ICR mice showed no biological reactivity, and the LD_50_ was more than 5,000 mg/kg b.w. by oral administration in both sexes of Wistar rats. In the *in vitro *study, the Jasmine flower extract exerted a vasorelaxation activity on the endothelium-intact aorta ring. The mechanisms probably involve the active component acting via the muscarinic receptors at the vascular endothelium and/or by stimulating nitric oxide release. Preliminary phytochemical analysis revealed that the flower of *J. sambac* contains antioxidants, coumarins, cardiac glycosides, essential oils, flavonoids, phenolics, saponins, and steroids, whereas alkaloids, anthraquinones, and tannins were not detected. Since the flower of *J. sambac* is tremendously employed in traditional medicines and in tea industries, the knowledge of the chemical compositions, toxicity, and pharmacological properties will provide insight information of this plant for its future application.

## Figures and Tables

**Table 1:** TLC Method.

Test	Standard	Mobile phase	Spraying reagent	Detection	Color
Alkaloids	Atropine	Toluene : ethyl acetate : dimethylamine (70 : 20 : 10)	Dragendorff reagent	Visible	Orange spot on yellow background
Anthraquinones	Rhein	Ethyl acetate : methanol : water (81 : 11 : 8)	KOH reagent	UV 366 nm	Yellow/orange/red
Antioxidants	Vit E	Ethyl acetate : toluene (50 : 50)	DPPH reagent	Visible	White spot on purple background
Coumarins	Coumarin	Toluene : ethyl acetate (90 : 10)	KOH reagent	UV 366 nm	Light blue/green
Essential oils	Eugenol	Toluene : ethyl acetate (93 : 7)	Anisaldehyde sulfuric acid reagent	Heat 100°C Visible	Purple/red
Flavonoids	Rutin	Ethyl acetate : formic acid : acetic acid : water (100 : 11 : 11 : 27)	Natural Product (NP/PEG)	UV 366 nm	Orange/yellow/green/blue
Cardiac glycosides	Ouabain	Ethyl acetate : methanol : water (81 : 11 : 8)	Kedde reagent	Visible	Pink/yellow/purple
Phenolics	Catechol	Toluene : ethyl acetate (97 : 3)	Ferric chloride reagent	Visible	Blue/black
Saponins	Saponin	Butanol : ethyl acetate : acetic acid : water (10.8 : 3.6 : 0.2 : 2.7)	Anisaldehyde sulfuric acid reagent	Visible	Blue
Tannins	Gallic acid	Metanol : ethyl acetate : acetic acid (10 : 90 : 0.1)	Ferric chloride reagent	Visible	Blue/black

**Table 2:** Phytochemical analysis of the ethanolic Jasmine flowers extract.

Compounds	Results
Alkaloids	**−**
Anthraquinones	**−**
Antioxidants	+
Coumarins	+
Cardiac glycosides	+
Essential oils	+
Flavonoids	+
Phenolics	+
saponins	+
steroids	+
tannins	**−**

**Table 3:** Systemic biological reactivity of the ethanolic Jasmine flower extract by intravenous injection.

Treatment	Weight (g)	Liver (g)	Kidney (g)	Spleen (g)	Heart (g)	Stomach (g)	Lung (g)	Pancreas (g)	Testis (g)
Group I: Control group	38.2 ± 0.66	2.882 ± 0.06	0.889 ± 0.01	0.158 ± 0.002	0.183 ± 0.004	0.229 ± 0.002	0.190 ± 0.005	0.290 ± 0.0005	0.287 ± 0.004
Group II: Treated group	37.0 ± 0.70	2.838 ± 0.04	0.890 ± 0.01	0.155 ± 0.002	0.177 ± 0.002	0.228 ± 0.002	0.193 ± 0.006	0.290 ± 0.0007	0.283 ± 0.003

Values are expressed as mean ± SEM (*n* = 5). Treated groups received a single dose of 15 mg of the extract via intravenous injection.

**Table 4:** Acute oral toxicity of the ethanolic Jasmine flower extract in male rats.

Male	Weight (g)	Liver (g)	Kidney (g)	Spleen (g)	Heart (g)	Stomach (g)	Lung (g)	Pancreas (g)	Testis (g)
Control (1% gum tragacanth)	318.4 ± 7.38	15.22 ± 0.43	2.70 ± 0.02	0.74 ± 0.03	1.196 ± 0.03	1.934 ± 0.02	1.580 ± 0.01	2.196 ± 0.02	3.726 ± 0.01
Jasmine flowers extract (5,000 mg/kg)	334.6 ± 10.64	14.47 ± 0.82	2.752 ± 0.02	0.744 ± 0.04	1.216 ± 0.05	1.924 ± 0.04	1.586 ± 0.01	2.226 ± 0.02	3.732 ± 0.02

Values are expressed as mean ± SEM (*n *= 5).

**Table 5:** Acute oral toxicity of the ethanolic Jasmine flower extract in female rats.

Female	Weight (g)	Liver (g)	Kidney (g)	Spleen (g)	Heart (g)	Stomach (g)	Lung (g)	Pancreas (g)	Uterus & Ovary (g)
Control (1% gum tragacanth)	237.6 ± 5.91	12.846 ± 0.76	1.978 ± 0.003	0.732 ± 0.001	0.996 ± 0.010	1.776 ± 0.002	1.472 ± 0.001	1.28 ± 0.004	0.916 ± 0.02
Jasmine flowers extract (5,000 mg/kg)	232 ± 5.11	12.788 ± 0.69	1.972 ± 0.01	0.728 ± 0.03	1.03 ± 0.05	1.716 ± 0.01	1.472 ± 0.003	1.282 ± 0.003	0.886 ± 0.012

Values are expressed as mean ± SEM (*n *= 5).
